# Sqstm1–GFP knock-in mice reveal dynamic actions of Sqstm1 during autophagy and under stress conditions in living cells

**DOI:** 10.1242/jcs.180174

**Published:** 2015-12-01

**Authors:** Atsushi Eino, Shun Kageyama, Takefumi Uemura, Hiromichi Annoh, Tetsuya Saito, Ichiei Narita, Satoshi Waguri, Masaaki Komatsu

**Affiliations:** 1 Department of Biochemistry, Niigata University Graduate School of Medical and Dental Sciences, Chuo-ku, Niigata 951-8510, Japan; 2 Division of Clinical Nephrology and Rheumatology, Niigata University Graduate School of Medical and Dental Sciences, Chuo-ku, Niigata 951-8510, Japan; 3 Department of Anatomy and Histology, Fukushima Medical University School of Medicine, Hikarigaoka, Fukushima 960-1295, Japan

**Keywords:** Sqstm1, Autophagy, Selective autophagy, Nrf2, Knock-in mice

## Abstract

Sqstm1 serves as a signaling hub and receptor for selective autophagy. Consequently, dysregulation of Sqstm1 causes imbalances in signaling pathways and disrupts proteostasis, thereby contributing to the development of human diseases. Environmental stresses influence the level of Sqstm1 by altering its expression and/or autophagic degradation, and also changes the localization of Sqstm1, making it difficult to elucidate the actions and roles of this protein. In this study, we developed knock-in mice expressing Sqstm1 fused to GFP (*Sqstm1-GFP^KI/+^*). Using these *Sqstm1-GFP^KI/+^* mice, we revealed for the first time the dynamics of endogenous Sqstm1 in living cells. Sqstm1–GFP was translocated to a restricted area of LC3-positive structures, which primarily correspond to the inside of autophagosomes, and then degraded. Moreover, exposure to arsenite induced expression of Sqstm1–GFP, followed by accumulation of the fusion protein in large aggregates that were degraded by autophagy. Furthermore, suppression of autophagy in *Sqstm1-GFP^KI/+^* mouse livers caused accumulation of Sqstm1–GFP and formation of GFP-positive aggregate structures, leading to severe hepatic failure. These results indicate that *Sqstm1-GFP^KI/+^* mice are a useful tool for analyzing Sqstm1 in living cells and intact animals.

## Abstract (precis)

**Highlighted Article:** We have developed *Sqstm1-GFP* knock-in mice, which are useful for elucidating the molecular mechanisms of cellular events, including autophagy, and various diseases in which Sqstm1 plays a role.

## INTRODUCTION

Sqstm1 (also known as p62) is conserved among metazoans, but not in plants or fungi. Sqstm1 contains multiple domains, including a Phox1 and Bem1p (PB1) domain, a zinc finger, two nuclear localization signals, a TRAF6-binding domain, a nuclear export signal, an LC3-interacting region (LIR), a Keap1-interacting region (KIR) and a ubiquitin-associated (UBA) domain, which mediate the interaction of the protein with various binding partners and also determine the cellular localization (Bitto et al., 2014; Ishii et al., 2013; Moscat and Diaz-Meco, 2009). Sqstm1 is a stress-inducible cellular protein; in particular, expression of the *Sqstm1* gene is positively regulated by the oxidative-stress responsive transcription factor nuclear factor erythroid 2 related factor 2 (Nrf2, also known as NFE2L2) (Ishii et al., 2000; Jain et al., 2010). Through its PB1 domain, Sqstm1 protein forms helical filaments (Ciuffa et al., 2015) that are translocated to sites of autophagosome formation (Itakura and Mizushima, 2011), where they serve as molecular templates for nucleation of the growing autophagosomal membrane (Ciuffa et al., 2015). At these sites, Sqstm1 eventually interacts with the autophagosome-localized protein LC3B (also known as MAP1LC3B; hereafter referred to as LC3) through its LIR, leading to its autophagic degradation (Ichimura et al., 2008; Pankiv et al., 2007; Shvets et al., 2008).

Sqstm1 also contributes to selective autophagy for ubiquitylated cargos (Bjorkoy et al., 2005; Rogov et al., 2014). In response to stressors, Sqstm1 is sequentially phosphorylated at Ser409 (corresponding to human Ser407) and Ser405 (corresponding to human Ser403) of the UBA domain, which increases the binding affinity of Sqstm1 for ubiquitin chains. As a result, Sqstm1 is translocated to autophagy substrates, such as ubiquitin-positive protein aggregates, damaged mitochondria and infecting bacterial cells (Lim et al., 2015; Matsumoto et al., 2015, 2011; Pilli et al., 2012). Recognition of the ubiquitin chain causes conversion from large helical filaments into shorter and less compact helical ones, which play a role in exclusive sequestration of ubiquitylated cargo in forming autophagosomes (Ciuffa et al., 2015). In addition, Sqstm1 self-oligomerizes in a PB1-domain-dependent manner to promote packaging of ubiquitylated cargos (Ichimura et al., 2008; Lamark et al., 2003; Pankiv et al., 2007). Meanwhile, Sqstm1 interacts with Nbr1, which has similar structural domains to those of Sqstm1 and serves as a receptor for selective autophagy through hetero-oligomerization mediated by PB1 domains (Kirkin et al., 2009). Interaction with LC3 is required for sufficient removal of ubiquitylated cargos during the process of selective autophagy (Bjorkoy et al., 2005; Ichimura et al., 2008).

Under selective autophagy-inducing conditions, mammalian target of rapamycin complex 1 (mTORC1) phosphorylates a specific serine residue (Ser351, corresponding to human Ser349) in KIR of Sqstm1 on the autophagic cargos (Ichimura et al., 2013). Phosphorylated Sqstm1 increases its binding affinity for Kelch-like ECH-associated protein 1 (Keap1), an adaptor of the ubiquitin ligase complex for Nrf2 and competitively abrogates the interaction between Nrf2 and Keap1. Consequently, Nrf2 translocates into the nucleus to induce the transcription of numerous cytoprotective genes encoding antioxidant proteins, detoxifying enzymes and multidrug transporters (Ichimura et al., 2013; Ishimura et al., 2014). Phosphorylated Sqstm1 and Keap1, together with autophagic cargos, are degraded by autophagy (Jain et al., 2015; Taguchi et al., 2012). This process enhances the positive-feedback loop resulting from Nrf2-mediated activation of *Sqstm1* gene expression (Jain et al., 2010), that is two major stress response pathways, selective autophagy and the Keap1–Nrf2 system, are coupled to each other through Ser351-phosphorylation of Sqstm1.

Recent studies of Sqstm1 have clarified its unique functions (Jiang et al., 2015; Moscat and Diaz-Meco, 2011; Rogov et al., 2014). However, given the diversity in characteristic properties of Sqstm1, such as stress-inducible expression, self-oligomerization, autophagic degradation and dynamic intracellular translocation, it is difficult to determine the roles of Sqstm1 in living cells and in tissues of intact animals. To overcome this issue, we developed *Sqstm1-GFP* knock-in mice and used them to investigate the dynamic features of Sqstm1 in cells and tissues under stress conditions.

## RESULTS

### Generation of *Sqstm1-GFP* knock-in mice

**Fig. 1.**
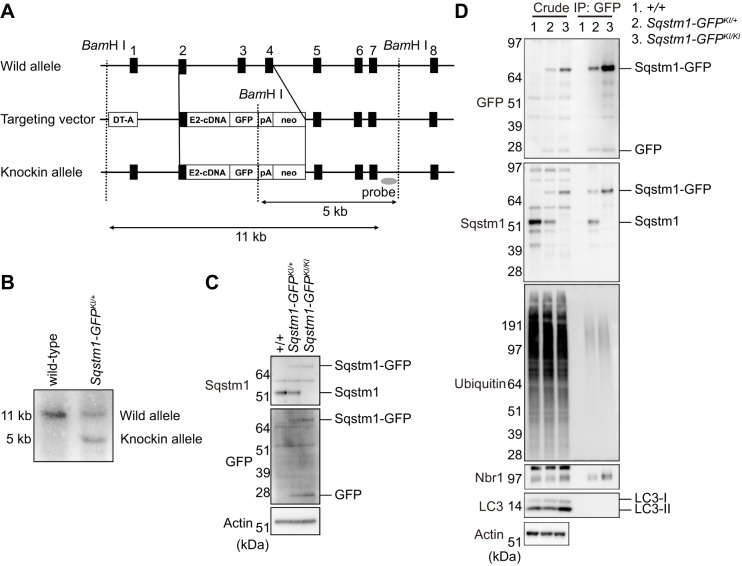
**Generation of *Sqstm1-GFP^KI/+^* mice.** (A) Schematic representation of the targeting vector and the targeted allele of the *Sqstm1* gene. The coding exons, numbered such that the initiation codon appears in exon 1, are depicted by black boxes. E2-cDNA-GFP-pA indicates the *Sqstm1* cDNA fragment (302–1326) fused with *GFP* cDNA and the SV40 poly(A) sequence. The probe used for Southern blot analysis is depicted as a gray ellipse. *Bam*HI, *Bam*HI sites; neo, neomycin-resistance gene cassette; DT-A, diphtheria toxin gene. (B) Southern blot analysis of genomic DNA extracted from mice tail snips. Wild-type and knock-in alleles were detected as 11- and 5-kb bands, respectively. (C) Immunoblot of Sqstm1 in MEFs. Lysates of MEFs of the indicated genotypes were immunoblotted with anti-Sqstm1, anti-GFP and anti-actin antibodies. LC3-I and LC3-II indicate a cytoplasmic form and an autophagosome-localized form of LC3B, respectively. The LC3-II on the inner membrane of autophagosomes is degraded after fusion of autophagosomes with lysosomes. (D) Interaction of Sqstm1–GFP with endogenous proteins. Lysates prepared from wild-type, *Sqstm1-GFP^KI/+^* and *Sqstm1-GFP^KI/KI^* MEFs were immunoprecipitated (IP) with anti-GFP antibody, followed by immunoblotting with the specified antibodies. The data shown are representative of three separate experiments.

**Fig. 2.**
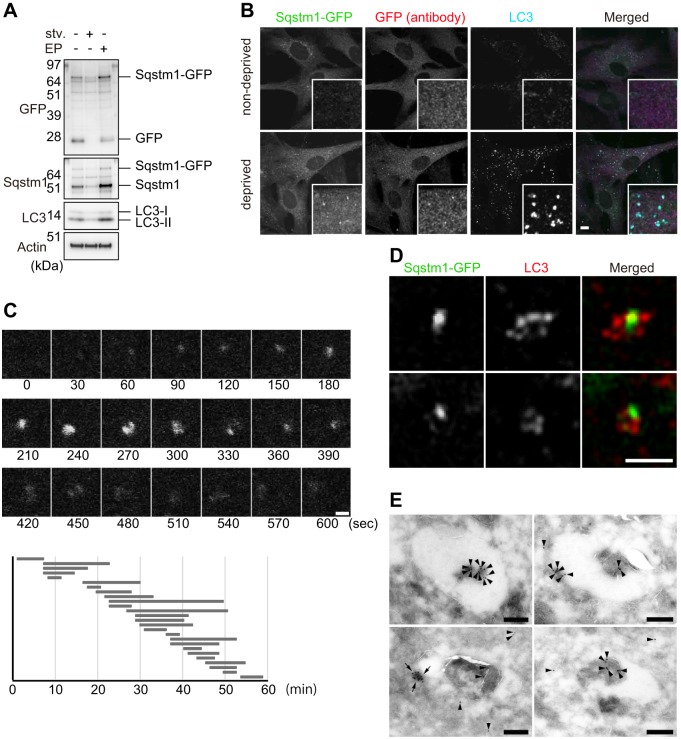
**Dissection of *Sqstm1-GFP^KI/+^* MEFs under starvation conditions.** (A) Immunoblot analysis. *Sqstm1-GFP^KI/+^* MEFs were cultured in regular or starvation medium for 2 h (stv.) or regular medium with E64d and pepstatin A for 24 h (EP). Cell lysates were prepared, followed by immunoblot analysis with the specified antibodies. The data shown are representative of three separate experiments. (B) Immunofluorescence staining. *Sqstm1-GFP^KI/+^* MEFs were cultured in regular (non-deprived) or starvation medium for 2 h (deprived), and then immunostained with anti-LC3 and anti-GFP antibodies. Each inset is a magnified image. Scale bar: 10 µm. (C) *Sqstm1-GFP^KI/+^* MEFs were cultured in starvation medium for 1 h and directly observed by time-lapse video microscopy. Scale bar: 1 μm. The duration of each spot of Sqstm1–GFP was measured for 26 cases, which is represented graphically underneath the images. (D) Structured Illumination Microscopic (SIM) analysis. *Sqstm1-GFP^KI/+^* MEFs under starvation conditions were immunostained with anti-LC3 antibody, and then observed by SIM. Scale bar: 1 µm. (E) *Sqstm1-GFP^KI/+^* MEFs were cultured in amino-acid-free medium for 1 h, and then fixed for immunoelectron microscopy using anti-GFP antibody as described in the Materials and Methods. Four representative profiles of autophagosomes are shown. Colloidal gold particles indicating Sqstm1–GFP (arrowheads) and high-density structures positive for Sqstm1–GFP (arrows) are labeled. Scale bars: 200 nm.

**Fig. 3.**
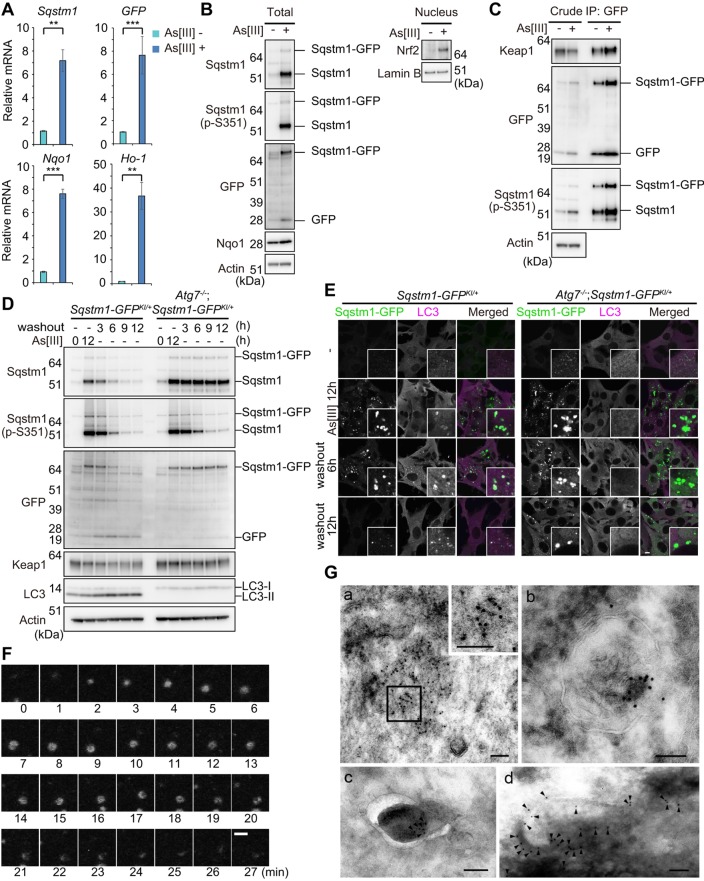
**Dissection of *Sqstm1-GFP^KI/+^* MEFs under stress conditions.** (A) Relative mRNA levels of *Sqstm1*, *GFP*, and *Nqo1* and *Ho-1*. Total RNAs were prepared from *Sqstm1-GFP^KI/+^* MEFs cultured for 12 h in the presence or absence of 10 µM sodium arsenite (As[III]) and then reverse-transcribed into cDNAs, which were used as templates for real-time quantitative PCR analysis. Values were normalized to the amount of each mRNA in the non-treated MEFs. The experiments were performed three times; data are mean±s.e.m. **P<0.01; ***P<0.001 (Welch test). (B) Immunoblot analysis. *Sqstm1-GFP^KI/+^* MEFs were cultured as described in A. Total cell lysates and nuclear fractions were prepared and subjected to immunoblot analysis with the specified antibodies. Data are representative of three independent experiments. (C) Immunoprecipitation (IP) analysis. *Sqstm1-GFP^KI/+^* MEFs were cultured as described in A. Immunoprecipitates obtained with anti-GFP antibody were analyzed by immunoblotting with the specified antibodies. Data are representative of three independent experiments. (D) Immunoblot analysis. *Sqstm1-GFP^KI/+^* and *Atg7^−/−^*;*Sqstm1-GFP^KI/+^* MEFs were challenged by As[III]. After removal of As[III], cells were cultured in regular medium for the indicated time. Cell lysates were prepared and subjected to immunoblot analysis with the specified antibodies. Data are representative of three independent experiments. (E) Immunofluorescence staining. *Sqstm1-GFP^KI/+^* MEFs were cultured as described in D, and then immunostained with anti-LC3 antibody. Each inset is a magnified image. Scale bar: 10 µm. (F) Time-lapse video microscopic analysis with *Sqstm1-GFP^KI/+^* MEFs cultured as shown in D. Scale bar: 1 μm. (G) Immunoelectron microscopy. *Sqstm1-GFP^KI/+^* MEFs were cultured as described in D, and fixed 1 h (d) or 3 h (a,b, and c) after removal of As[III]. They were then immunolabeled with anti-GFP antibody, followed by secondary antibody conjugated to colloidal gold particles (indicated by arrowheads for c and d). The boxed region in a was magnified and is shown in the inset. For better recognition of colloidal gold particles on dark background area, gamma correction was performed for c and d. Scale bars: 100 nm.

To monitor dynamics of Sqstm1 during autophagy *in vivo*, we generated knock-in mice that express Sqstm1 C-terminally fused to GFP (Sqstm1–GFP) (Fig. 1A). We verified germ-line transmission of the knock-in (*Sqstm1-GFP^KI/+^*) by Southern blot analysis (Fig. 1B). *Sqstm1-GFP^KI/+^* mice were fertile and showed no obvious pathological phenotypes for at least 2 years. To test the expression level of Sqstm1–GFP, we isolated mouse embryonic fibroblasts (MEFs) from wild-type, *Sqstm1-GFP^KI/+^*, and *Sqstm1-GFP^KI/KI^* embryos and immortalized them by introducing simian virus 40 (SV40) T (large T) antigen. Immunoblot analysis with anti-Sqstm1 antibody revealed that *Sqstm1-GFP^KI/+^* MEFs expressed both Sqstm1–GFP and Sqstm1 (Fig. 1C), whereas wild-type MEFs expressed only Sqstm1, and *Sqstm1-GFP^KI/KI^* MEFs expressed only Sqstm1–GFP (Fig. 1C). Next, to determine whether GFP-tagging of Sqstm1 affected its ability to bind endogenous proteins, we performed immunoprecipitation assays with anti-GFP antibody. Sqstm1–GFP from *Sqstm1-GFP^KI/+^* MEFs formed a complex with endogenous Sqstm1 (Fig. 1D), and Sqstm1–GFP in *Sqstm1-GFP^KI/+^* and *Sqstm1-GFP^KI/KI^* MEFs had the ability to interact with endogenous ubiquitylated proteins and Nbr1 (Fig. 1D). We hardly detected any LC3 signal in immunoprecipitates prepared from *Sqstm1-GFP^KI/+^* and *Sqstm1-GFP^KI/KI^* MEFs (Fig. 1D), probably due to their transient interaction at autophagosome formation site and rapid degradation through autophagy (Itakura and Mizushima, 2011). However, Sqstm1–GFP extensively colocalized with LC3-positive structures and was then degraded (see Figs 2 and 3). On the basis of these results, we concluded that GFP tagging did not influence the interactions with endogenous proteins, at least in regard to autophagy-related proteins.

### Dynamics of Sqstm1–GFP during starvation-induced autophagy

In the next series of experiments, we investigated Sqstm1-GFP in *Sqstm1-GFP^KI/+^* MEFs under nutrient-deprived conditions. Upon nutrient deprivation of *Sqstm1-GFP^KI/+^* MEFs, the levels of Sqstm1-GFP and Sqstm1 decreased (Fig. 2A). Free GFP, which is derived from degradation of Sqstm1-GFP in lysosomes (Ishimura et al., 2014), disappeared in response to nutrient deprivation (Fig. 2A). Treatment of the MEFs with lysosomal enzyme inhibitors, E64d and pepstatin A increased levels of Sqstm1–GFP and Sqstm1, as well as of LC3-II (Fig. 2A), but decreased the level of free GFP (Fig. 2A). These results imply that Sqstm1–GFP is subject to autophagic degradation. Fluorescence microscopic analysis revealed that upon nutrient deprivation, structures positive for Sqstm1–GFP with a diameter ranging from 0.5 to 1 μm appeared in the cytoplasm (Fig. 2B). Immunofluorescence analysis with anti-LC3 antibody revealed that the number of LC3-positive puncta dramatically increased upon nutrient deprivation and 20.7±2.5% (mean±s.e.m., *n*=30 cells) of these puncta were colocalized with Sqstm1–GFP (Fig. 2B). These results corresponded approximately to those obtained upon immunofluorescence staining of endogenous Sqstm1 with a specific antibody (Bjorkoy et al., 2005; Komatsu et al., 2007). To directly determine the lifespan of these Sqstm1–GFP puncta in *Sqstm1-GFP^KI/+^* MEFs, we carried out time-lapse video microscopic analysis. In response to nutrient deprivation, the Sqstm1–GFP dots appeared at random in the cytosol, persisted for a time, and then disappeared. The mean (±s.e.m.) duration of this process was 9.49±6.46 min (Fig. 2C; Movie 1), similar to the life-time of the autophagosome (Mizushima et al., 2001). To explore the localization of Sqstm1–GFP on the membrane structures more precisely in cells, we first carried out analysis using structured illumination microscopy (SIM). SIM revealed cup-shaped LC3 structures, in which the LC3 intensity differed among sites, and Sqstm1–GFP translocated onto restricted parts of LC3-positive structures (Fig. 2D). Next, we performed immunoelectron microscopy analyses with anti-GFP antibody. Sqstm1–GFP was localized both inside and on the restricted part of the outer membranes of autophagosomes. Counting of colloidal gold particles indicated that the majority of the signal (74.5±38.8%; mean±s.d., *n*=20) was in the engulfed region (Fig. 2E). Aggregate-like structures positive for Sqstm1–GFP were occasionally found on the autophagosomal membranes (Fig. 2E).

### Dynamics of Sqstm1–GFP under stress conditions

Consistent with a previous report (Ichimura et al., 2013), gene expression of *Sqstm1* in *Sqstm1-GFP^KI/+^* MEFs was dramatically induced upon exposure to sodium arsenite (As[III]) (Fig. 3A). Likewise, treatment of the MEFs with As[III] induced expression of *Sqstm1-GFP* (Fig. 3A). We confirmed prominent upregulation of Sqstm1 and Sqstm1–GFP proteins, both of which were phosphorylated at Ser351, in response to As[III] (Fig. 3B). Immunoprecipitation assay with anti-GFP antibody revealed that As[III] treatment significantly enhanced the interaction between Keap1 and Sqstm1–GFP (Fig. 3C). Thus, the exposure of As[III] was accompanied by nuclear accumulation of Nrf2, as well as induction of Nrf2 targets such as Nqo1 and Ho-1 (also known as HMOX1) (Fig. 3A,B). The properties of Sqstm1–GFP and the Keap1–Nrf2 pathway in *Sqstm1-GFP^KI/+^* MEFs were consistent with those in wild-type MEFs (Ichimura et al., 2013; Lau et al., 2010). As with non-tagged endogenous Sqstm1, the levels of Sqstm1–GFP and its phosphorylated form in *Sqstm1-GFP^KI/+^* MEFs decreased 6 h after removal of As[III], whereas levels of LC3-II increased (Fig. 3D), suggesting autophagic turnover of Sqstm1–GFP. No such downregulation was observed in the case of *Atg7^−/−^*;*Sqstm1-GFP^KI/+^* MEFs (Fig. 3D), in which autophagy is impaired (Komatsu et al., 2005). Remarkably, removal of As[III] decreased levels of Ser351-phosphorylated Sqstm1 even in *Atg7^−/−^*;*Sqstm1-GFP^KI/+^* MEFs (Fig. 3D), suggesting that there are phosphatase(s) that recognize Ser351-phosphorylated Sqstm1.

Loss of autophagy in mouse tissues is usually accompanied by formation of large aggregate structures positive for Sqstm1, in particular in quiescent cells such as neurons and hepatocytes (Komatsu et al., 2007). Likewise, immunofluorescence analysis revealed aggregates positive for Sqstm1–GFP in *Atg7^−/−^*;*Sqstm1-GFP^KI/+^* MEFs (Fig. 3E), but the number and size were obviously less and smaller than those in *Atg7*-deficient hepatocytes and neurons. This result might be accounted for by dilution of aggregate structures by rapid cell division of MEFs. Upon exposure of As[III], aggregate structures positive for Sqstm1–GFP with a diameter ranging from 1 to 5 μm formed in the cytoplasm of both *Sqstm1-GFP^KI/+^* and *Atg7^−/−^*;*Sqstm1-GFP^KI/+^* MEFs (Fig. 3E). Most of these structures in *Sqstm1-GFP^KI/+^* MEFs contained LC3, whereas the aggregates in *Atg7^−/−^*;*Sqstm1-GFP^KI/+^* MEFs hardly contained any (Fig. 3E). Both the number and size of such structures in *Sqstm1-GFP^KI/+^*, but not *Atg7^−/−^*;*Sqstm1-GFP^KI/+^* MEFs, decreased following washout of As[III] in a time-dependent manner (Fig. 3E). Time-lapse video microscopic analysis revealed the dynamics of Sqstm1–GFP under stress conditions and in the recovery state (Movies 2–5). Aggregate structures positive for GFP appeared in *Sqstm1-GFP^KI/+^* MEFs after 6 h of As[III] treatment, and both number and size increased with time (Movie 2). In the case of *Atg7^−/−^*;*Sqstm1-GFP^KI/+^* MEFs, such structures were formed sooner (Movie 3). Most of these structures in *Sqstm1-GFP^KI/+^* MEFs persisted for several hours after removal of As[III], and they frequently became small aggregates (<1 μm) by 4 h after the removal. Subsequently, several small aggregates became crescent-like structures and eventually disappeared (Fig. 3F; Movie 4). Even after 12 h after the removal of As[III], a small number of large aggregate structures was still observed (Fig. 3F; Movie 4). By contrast, most large aggregate structures in *Atg7^−/−^*;*Sqstm1-GFP^KI/+^* MEFs remained even 12 h after the removal of As[III] (Movie 5). Immunoelectron microscopy indicated that Sqstm1–GFP in *Sqstm1-GFP^KI/+^* MEFs localized in aggregated structures (Fig. 3Ga) and autolysosomal structures (Fig. 3Gb), and was occasionally associated with phagophore profiles (Fig. 3Gc,d). Taken together, we conclude that the *Sqstm1-GFP^KI/+^* MEFs immortalized by SV40 T-antigen are available for dynamic analysis of Sqstm1 during starvation- and stress-induced autophagy. We note that because the SV40 T-antigen targets multiple cellular pathways, primary cultured cells derived from *Sqstm1-GFP^KI/+^* mice should be used to explore a role of Sqstm1 in signal transduction pathways (e.g. atypical PKC, ERK1, NF-κB and caspase-8) in which Sqstm1 serves as a signaling hub (Moscat and Diaz-Meco, 2011).

### Sqstm1–GFP in autophagy-deficient tissues

**Fig. 4.**
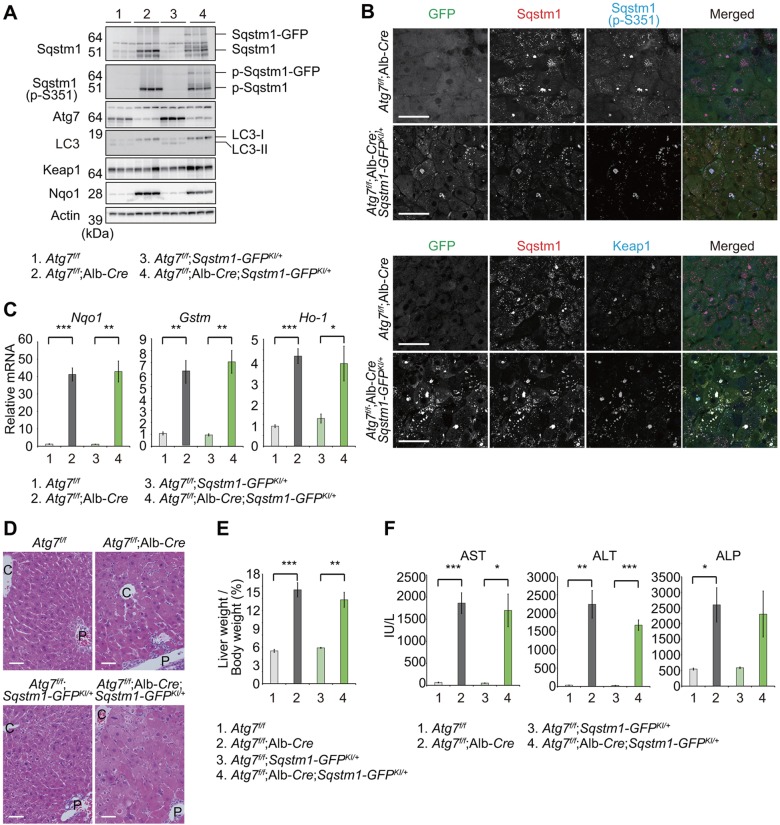
***In vivo* analyses of *Sqstm1-GFP^KI/+^* mice under autophagy-impaired conditions.** (A) Immunoblotting of livers of mice of the indicated genotypes. Liver homogenates prepared from 4–5-week-old mice of the indicated genotypes were subjected to NuPAGE and analyzed by immunoblotting with the indicated antibodies. (B) Immunohistofluorescence analysis of Sqstm1–GFP, Sqstm1, Ser351-phosphorylated Sqstm1 and Keap1. Liver sections from 4–5-week-old mice of the indicated genotypes were triple-immunostained for GFP, Sqstm1, and Ser351-phosphorylated Sqstm1 (upper panels) or GFP, Sqstm1 and Keap1 antibodies (bottom panels). The rightmost column shows the merged images of GFP (green), Sqstm1 (red), and S351-phosphorylated Sqstm1 (blue) in the upper panel, and GFP (green), Sqstm1 (red), and Keap1 (blue) in the bottom panel. Scale bars: 50 µm. (C) Quantitative real-time PCR analyses of *Nqo1*, *Gstm1*, and *Ho-1* in mouse livers. Total RNAs were prepared from livers of 4–5-week-old mice of the indicated genotypes. Values were normalized to the amount of mRNA in the *Atg7^f/f^* liver. Experiments were performed three times. Data are means±s.e.m. *P<0.05, **P<0.01, ***P<0.001 (Welch test). (D) Histological analysis of livers from 4–5-week-old mice of the indicated genotypes. Livers were processed for H&E staining. C, central vein, P, portal vein. Scale bars: 50 µm. (E) Liver weight normalized to body weight. Data are mean±s.e.m. of *Atg7^f/f^* (*n*=4), *Atg7^f/f^*;Albumin-*Cre* (*n*=6), *Atg7^f/f^*;*Sqstm1-GFP^KI/+^*(*n*=4) and *Atg7^f/f^*;Albumin-*Cre*;*Sqstm1-GFP^KI/+^* (*n*=5) mice from each group. **P<0.01, ***P<0.001 (Welch test). (F) Liver function tests of the mice used in E. Serum levels of aspartate aminotransferase (AST), alanine aminotransferase (ALT) and alkaline phosphatase (ALP) were measured. Data are mean±s.e.m. *P<0.05, **P<0.01, ***P<0.001 (Welch test).

Finally, we tested the utility of *Sqstm1-GFP* knock-in mice for *in vivo* studies in intact animals. To this end, we crossbred the *Sqstm1-GFP^KI/+^* with hepatocyte-specific *Atg7*-knockout mice (*Atg7^f/f^*;Albumin-*Cre*) (Komatsu et al., 2007), because the pathogenic roles of Sqstm1 have been extensively investigated in mice with liver-specific defects in autophagy (Komatsu et al., 2010, 2007; Ni et al., 2014; Takamura et al., 2011). Like endogenous Sqstm1, Sqstm1–GFP and its phosphorylated form prominently accumulated in livers of *Atg7^f/f^*;Albumin-*Cre*;*Sqstm1-GFP^KI/+^* mice (Fig. 4A). Immunohistofluorescence revealed aggregate structures positive for Sqstm1–GFP with a diameter ranging from 1 to 10 µm in the livers of the mutant mice (Fig. 4B). These aggregates were also colabeled by antibodies against Sqstm1, Ser351-phosphorylated Sqstm1, and/or Keap1 (Fig. 4B). Their size and number, as well as composition, were comparable to those of aggregates formed in livers of *Atg7^f/f^*;Albumin-*Cre* (Fig. 4B).

Persistent activation of Nrf2 due to sequestration of Keap1 into Sqstm1-positive structure causes hepatomegaly and liver failure in *Atg7^f/f^*;Albumin-*Cre* and *Atg5^f/f^*;Albumin-*Cre* mice (Komatsu et al., 2010; Ni et al., 2014). Therefore, we investigated whether GFP-tagging of Sqstm1 affected the liver phenotypes of *Atg7^f/f^*;Albumin-*Cre*. Gene expression of Nrf2 targets, including *Nqo1*, *Gstm* and *Ho-1*, was significantly induced in livers of *Atg7^f/f^*;Albumin-*Cre*;*Sqstm1-GFP^KI/+^*, and the induced levels were quite similar to those in *Atg7^f/f^*;Albumin-*Cre* livers (Fig. 4C). As in *Atg7^f/f^*;Albumin-*Cre* livers, the level of Nqo1 protein was elevated in *Atg7^f/f^*;Albumin-*Cre*;*Sqstm1-GFP^KI/+^* livers (Fig. 4A). Hematoxylin & eosin (H&E) staining revealed hepatocytic swelling, as well as infiltration of inflammatory cells, in both *Atg7^f/f^*;Albumin-*Cre*;*Sqstm1-GFP^KI/+^* and *Atg7^f/f^*;Albumin-*Cre* livers (Fig. 4D). As predicted, *Atg7^f/f^*;Albumin-*Cre*;*Sqstm1-GFP^KI/+^* mice exhibited liver enlargement and leakage of hepatic enzymes (Fig. 4E,F), and there was no statistical difference in the extent of damage between *Atg7^f/f^*;Albumin-*Cre*;*Sqstm1-GFP^KI/+^* and *Atg7^f/f^*;Albumin-*Cre* mice (Fig. 4E,F). These results indicate that *Sqstm1-GFP^KI/+^* mice are useful for *in vivo* analysis in intact animals, at least under conditions in which autophagy is impaired.

## DISCUSSION

In this study, we developed a line of mice that express GFP-fused Sqstm1 under the control of the *Sqstm1* promoter, and demonstrated that Sqstm1–GFP in *Sqstm1-GFP^KI/+^* mice and derived cells closely resembles the properties of endogenous Sqstm1. Dysfunction of Sqstm1 is involved in the pathogenesis of human diseases. For example, various mutations of *Sqstm1* have been identified in patients with Paget disease of bone (PDB), amyotrophic lateral sclerosis (ALS) and frontotemporal lobar degeneration (FTLD) (Rea et al., 2014). Moreover, aberrant accumulation of Sqstm1-positive aggregated structures has been detected in patients with liver disorders including non-alcoholic steatohepatitis and α1-antitrypsin deficiency, tumors such as hepatocellular carcinoma and various neurodegenerative diseases (Zatloukal et al., 2002). Furthermore, *Sqstm1* has been identified as a pathogenic target of 5q copy number gains in kidney cancer (Li et al., 2013). Thus, the *Sqstm1-GFP^KI/+^* mice could become useful tools for elucidating the pathophysiological roles of Sqstm1 in the aforementioned human diseases by crossbreeding them with disease-related model mice. At the molecular level, it remains unknown how Sqstm1 is selectively degraded by autophagy and how it regulates multiple signaling pathways. For example, it is unclear how Sqstm1 is translocated to sites of autophagosome formation prior to the formation of membranes, and how Sqstm1 asymmetrically localizes on the inner membrane of the autophagosome. Our *Sqstm1-GFP^KI/+^* cells should be useful tools for addressing such fundamental questions.

## MATERIALS AND METHODS

### Cell culture and knockdown

Mouse embryonic fibroblasts (MEFs) were prepared as described previously (Komatsu et al., 2007). Immortalized MEFs were established by infecting MEFs with a recombinant retrovirus carrying a temperature-sensitive simian virus 40 large T antigen. MEFs were grown in Dulbecco's modified Eagle's medium (DMEM) containing 10% fetal bovine serum (FBS), 5 U/ml penicillin, and 50 µg/ml streptomycin. We used Earle's balanced salt solution (EBSS) as a starvation medium. Sodium arsenite was purchased from Wako Pure Chemical Industries (Osaka, Japan). E64d and Pepstatin A were from Peptide Institute, Inc (Osaka, Japan).

### Mice

The targeting vector for *Sqstm1-GFP* knock-in mice was constructed by inserting the cDNA fragment encoded by exons 3–8 (302–1326) of *Sqstm1*, the *GFP* cDNA, and a poly(A) signal sequence after exon 2 of the endogenous *Sqstm1* gene (Fig. 1A). A neo resistance gene cassette (*mc1-neo-pA*) was ligated behind the polyA signal sequence. We electroporated the targeting vector into mouse TT2 embryonic stem cells, selected with G418 (250 µg/ml; Thermo Fisher Scientific), and then screened for homologous recombinants by Southern blot analyses. Southern blots were performed by digestion of genomic DNA with *BamH*I and hybridization with the probe shown in Fig. 1A. Genotyping of mice by PCR was performed using the following primers: 5′-TGCACCCCAATGTGATCTGTGATGGTTGCA-3′, and 5′-TCCCCTGCACGAGGAGGACGTGGGCTCCAG-3′. *Atg7^f/f^*;Albumin-*Cre* mice used in this study were described previously by our group (Komatsu et al., 2007). Mice were housed in specific pathogen-free facilities, and the Ethics Review Committee for Animal Experimentation of Niigata University School of Medicine approved the experimental protocols.

### Immunological analysis

Livers were homogenized in 0.25 M sucrose, 10 mM HEPES pH 7.4 and 1 mM dithiothreitol (DTT). Cells were lysed with ice-cold TNE buffer (10 mM Tris-HCl pH 7.5, 1% Nonidet P-40, 150 mM NaCl, 1 mM EDTA, and protease inhibitors). Nuclear fractions from cells were prepared using the NE-PER nuclear and cytoplasmic extraction reagents (Thermo Fisher Scientific). Samples were separated using a NuPAGE system (Thermo Fisher Scientific) on 4–12% Bis-Tris gels in MOPS-SDS buffer, and then transferred to a polyvinylidene difluoride (PVDF) membrane. Antibodies against GFP (A-6455, Thermo Fisher Scientific), LC3B (#2775, Cell Signaling Technology), Nqo1 (ab34173, Abcam), Nrf2 (H-300, Santa Cruz Biotechnology), Keap1 (10503-2-AP, Proteintech Group, Chicago, IL), Nbr1 (MKA0049AF, ProteinExpress, Chiba, Japan), ubiquitin (sc-8017, Santa Cruz Biotechnology), actin (MAB1501R, Merck Millipore), and lamin B (M-20, Santa Cruz Biotechnology) were purchased from the indicated suppliers. Antibodies against Sqstm1 (Komatsu et al., 2007) and Ser351-phosphorylated Sqstm1 (Ichimura et al., 2013) were as described previously.

### Immunocytochemistry

Cells grown on coverslips were fixed in 4% paraformaldehyde in PBS for 15 min, permeabilized with 50 µg/ml digitonin for 10 min or 0.1% Triton X-100 in PBS for 5 min, blocked with 0.1% (w/v) gelatin in PBS for 30 min, and then incubated overnight with primary antibodies against GFP (A-6455, Thermo Fisher Scientific) and LC3B (#2775, Cell Signaling Technology). After washing, cells were incubated with Alexa-Fluor-conjugated goat anti-rabbit-IgG and anti-mouse-IgG secondary antibodies (Thermo Fisher Scientific) for 60 min. Cells were imaged using a confocal laser-scanning microscope (FV1000, Olympus, Inc., Tokyo, Japan) with a UPlanSApo 100× NA 1.40 oil objective lens. *z*-projection stack images were acquired with *z* steps of 0.5 µm. SIM images were acquired on a Zeiss ELYRA S.1 microscope equipped with a Plan Apochromat oil-immersion objective (63×, 1.4 NA, Carl Zeiss). Image contrast and brightness were adjusted using Photoshop CS4 (Adobe).

### Real-time quantitative PCR analysis

Using a Transcriptor First-Strand cDNA synthesis kit (Roche Applied Science), cDNA was synthesized from 1 µg of total RNA. Quantitative PCR was performed using LightCycler^®^ 480 Probes Master mix (Roche Applied Science) on a LightCycler^®^ 480 (Roche Applied Science). Signals were normalized to the corresponding levels of *Gusb* mRNA (encoding β-glucuronidase). The sequences of the primers used were: *Sqstm1* left, 5ʹ-GCTGCCCTATACCCACATCT-3ʹ; *Sqstm1* right, 5ʹ-CGCCTTCATCCGAGAAAC-3ʹ; *GFP* left, 5ʹ-CAGCAGAACACCCCCATC-3ʹ; *GFP* right, 5ʹ-TGGGTGCTCAGGTAGTGGTT-3ʹ; *Nqo1* left, 5ʹ-AGCGTTCGGTATTACGATCC-3ʹ; *Nqo1* right, 5ʹ-AGTACAATCAGGGCTCTTCTCG-3ʹ; *Gstm* left, 5ʹ-CTACCTTGCCCGAAAGCAC-3ʹ; *Gstm* right, 5ʹ-ATGTCTGCACGGATCCTCTC-3ʹ; *Ho-1* left, 5ʹ-GGTCAGGTGTCCAGAGAAGG-3ʹ; *Ho-1* right, 5ʹ-CTTCCAGGGCCGTGTAGATA-3ʹ; *Gusb* left, 5ʹ-GATGTGGTCTGTGGCCAAT-3ʹ and *Gusb* right, 5ʹ-TGTGGGTGATCAGCGTCTT-3ʹ.

### Histological examination

Fixation and embedding procedures for immunohistofluorescence were as described previously (Waguri and Komatsu, 2009). Briefly, mouse livers were quickly excised, cut into small pieces, and then fixed by immersing in 4% paraformaldehyde with 4% sucrose in 0.1 M phosphate buffer, pH 7.4. After rinsing, the livers were embedded in paraffin for staining with H&E and in OCT compound for immunohistofluorescence. For immunolabeling, sections were processed for antigen retrieval in Immunosaver (Nissin EM, Tokyo, Japan) using a microwave processor (Azumaya Corporation, Tokyo, Japan), followed by blocking and incubation for 2–3 days at 4°C with three primary antibodies; mouse monoclonal antibody against GFP (clone 7.1/13.1, Roche), guinea pig polyclonal antibody against Sqstm1 (GP62-C, Progen Biotechnik, Heidelberg, Germany), and rabbit polyclonal antibody against Ser351-phosphorylated Sqstm1 (Ichimura et al., 2013) or Keap1 (Proteintech Group). Secondary antibodies were Alexa-Fluor-488-conjugated donkey anti-mouse-IgG (Jackson ImmunoResearch laboratories), DyLight-549-conjugated donkey anti-guinea-pig-IgG (Jackson ImmunoResearch laboratories) and DyLight-649-conjugated donkey anti-rabbit-IgG (Jackson ImmunoResearch laboratories) antibodies. Immunofluorescence images were acquired with a laser scanning confocal microscope (FV1000, Olympus) with a 40× objective lens (UPlanSApo, oil, NA 1.3, Olympus). After image acquisition, contrast and brightness were adjusted using Photoshop CS4 (Adobe).

### Electron microscopic analysis

*Sqstm1-GFP^KI/+^* MEFs were cultured in amino-acid-free medium for 1 h, then fixed with 0.1 M phosphate buffer pH 7.4 containing 4% paraformaldehyde and 0.03–0.06% glutaraldehyde. In the experiment using As[III], the cells were fixed with 0.1 M phosphate buffer pH 7.4 containing 4% paraformaldehyde alone. Ultrathin cryosections were prepared and immunolabeled with anti-GFP antibody (Abcam), followed by incubation with secondary antibody conjugated with colloidal gold particles (12-nm diameter; Jackson ImmunoResearch laboratories). Detailed procedures used for cell freezing, sectioning and immunoreactions are as given previously (Waguri and Komatsu, 2009). Sections were viewed using an electron microscope (JEM1200EX, JEOL, Tokyo, Japan). In some electron microscopy images, gamma correction was performed using the Adobe Photoshop software for better recognition of colloidal gold particles on dark background area, as indicated in the figure legend.

### Statistical analysis

Values, including those displayed in the graphs, are mean±s.e.m. or mean±s.d., as indicated. Statistical analysis was performed using the unpaired *t*-test (Welch test). *P*<0.05 denoted statistical significance.
